# Editorial

**Published:** 1841-01-30

**Authors:** 


THE MEDICAL EXAMINER.

PHILADELPHIA, JANUARY 30, 1841.

### HYDROSUDOPATHY AND THE PEASANT PRIESSNITZ.

The thirty-third number of our last volume* contained a review of Dr. Bigel's Manual of Hydrosudopathy, from which we condensed an account of the treatment of diseases by cold water, sweating, exercise, and regimen, practised by a certain Priessnitz, at Graeffenberg. The subject deserves, and has excited some attention in the medical journals. We notice an account of Dr. Bigel's Manual in the October number of the Medico-Chirurgical Review, which is copied into the January number of the American Journal of the Medical Sciences. The London Review, however, and the American copyist, have fallen into an error about Herr Priessnitz, in dubbing him Doctor.—Priessnitz is an uneducated peasant, who has no therapeutic pretensions beyond cold water and sweating. He has had the sagacity to turn to account two old and familiar remedies, and, by pushing them to an extent hitherto untried, has been able, we doubt not, in many cases, to produce very powerful effects upon the system. The hint is worth the attention of the profession, emanating, though it does, from a source without claim to the university honours bestowed by our cotemporaries.

### EXPERIMENTS UPON THE BODIES OF CRIMINALS.

We published in our last number a report of the experiments performed on the body of Morris, with the post mortem examination. The bodies of two other criminals have been submited to the same experiments during the last

* August 15, 1840.

three years.* A brief review of these three cases may not be uninteresting. An account of the galvanic experiments in the last, will probably be supplied by the able experimenters themselves; we merely allude to the subject cursorily, to state that, while they served to confirm the results furnished in the two first cases, they elicited no new fact; they merely corroborated laws already well established. These laws as deducible from the experiments upon Williams and Koebler, we believe, may be thus stated:

So long as a nerve retains its independent vitality, a galvanic current transmitted along its course will produce contraction of the muscles to which it is distributed; this, however, is only absolutely true of the nerves which, during life, were under the control of the will: the muscles of organic life, the heart, and muscular plane of the intestinal tube, remain unaffected.

This current is in all cases transmitted more readily, and with more striking results, through the medium of the skin, than by a direct contact of the poles with the nerve itself.

The effects are more marked when the current is transmitted from the peripheric extremity of a nerve towards its centre.

With these laws in view, we can almost establish *a priori* the result of any experiments which may be proposed, and when these, as in the case of Morris, do not vary essentially from such as have already been tried, the results, as we might anticipate, will be found identical.

The period during which the nerve retains its susceptibility to galvanic influences, varies very much in different individuals; the action of the current appears to abridge it: thus, when one limb has become insensible from the repetition of experiments, the corresponding limb may still be made to act with vigour. Previous cerebral exhaustion, the result of remorse or contrition, may also have its effect, as well as the particular state of the atmosphere, upon the suspended body: it is certain that, in the case of the last criminal, upon whom these influences may be supposed to have operated, the susceptibility was from the commencement much less marked than is usual in bodies similarly constituted, and was lost at a much earlier period.

A difference of opinion exists in regard to the propriety of collecting the symptoms presented during the stage of suspension; this can only be resolved by deciding the question of its practical utility. Certainly, in the cases which we are now considering, they remain, for the moment, isolated facts, by which we cannot immediately profit; but who shall decide that they may not, at some future period, serve to connect some chain of evidences illustrative of a physiological law? as facts, they cannot be devoid of value, but is their importance commensurate with the painful sacrifice of feeling, the forced stoicism of the physician appointed to collect them? With a utility so vague as in the above instances, we think not. But when taken with a view to decide an important and obscure medico-legal inquiry, viz., the signs of death by suspension and the means of distinguishing between a suicidal and homicidal death thus produced, their importance is too evident to require commentary. The oracles of our profession upon this branch* (medical jurisprudence) have not hesitated to take the evidence of executioners, to hang dead bodies, in order, by subsequent examination, to detect the nature of the lesions produced; in fact, to collect all, the most minute, evidence capable of throwing light upon a point of such interest to society in general.

We will resume this subject in our next.

* See Select Medical Library, No. 12, Oct. 1839, and American Journal Medical Sciences, No. 51, May, 1840.

* See Medical Examiner, Vol. 3, No. 46.

